# Prevention of Early-Onset Colorectal Cancer: Not One Size Fits All

**DOI:** 10.1093/jncics/pkab030

**Published:** 2021-05-20

**Authors:** Ebunoluwa E Otegbeye, Graham A Colditz, Yin Cao

**Affiliations:** 1 Department of Surgery, Washington University School of Medicine, St. Louis, MO, USA; 2 Division of Public Health Sciences, Department of Surgery, Washington University School of Medicine, St. Louis, MO, USA; 3 Alvin J. Siteman Cancer Center, Washington University School of Medicine, St. Louis, MO, USA; 4 Division of Gastroenterology, Department of Medicine, Washington University School of Medicine, St. Louis, MO, USA

The incidence of early-onset colorectal cancer (CRC), commonly defined as diagnosis prior to the age of 50 years, has been increasing across the globe (1-5). In the United States, in sharp contrast to the decline in CRC diagnosed among adults older than 55 years, early-onset CRC has been increasing at 2% each year with an estimated 18 000 new diagnoses in 2020 (1). Approximately only 16%-20% of early-onset CRCs are attributable to hereditary or genetic syndromes (6,7); therefore, examining nongenetic risk factors and contributors to the rising incidence is a priority. Risk factors such as obesity, smoking, alcohol use, and low-fiber diet have been linked with increased CRC risk in studies that primarily recruited adults older than 50 years (6). Because of the low incidence of early-onset CRC (7.7 per 100 000 persons in the United States) (1) and a limited number of large population-based studies among younger adults, elucidating the role of putative CRC risk factors in early onset CRC remains a research challenge (7).

In this issue of the Journal, Archambault and colleagues (8) harness the power of 3 international consortia (Colon Cancer Family Registry, the Colorectal Transdisciplinary Study [CORECT], and the Genetics and Epidemiology of Colorectal Cancer Consortium [GECCO]), including 10 case-control and 3 cohort studies, to examine the role of well-established CRC risk factors in early-onset CRC. The study population consisted of 3767 early-onset CRC and 4049 CRC-free controls of European descent from countries with a similar rising incidence of early-onset CRC, including the United States, the United Kingdom, Canada, Sweden, and Germany. The authors examined 16 self-reported anthropometric, lifestyle, dietary, and pharmacological risk factors ascertained at patient enrollment and/or blood collection for cohort study or 1-2 years prior to selection for case-control studies. A total of 23 437 late-onset CRCs (aged 50 years or older) and 35 311 CRC-free controls were also included for effect size comparisons.

Overall, the following factors were associated with modestly increased risk of early-onset CRC: alcohol consumption of more than 28 g/day (odds ratio [OR] = 1.25, 95% confidence interval [CI] = 1.04 to 1.50) or abstinence (OR = 1.23, 95% CI = 1.08 to 1.39) compared with 1-28 g/day, high red meat intake (per sex- and study-specific quartile) compared with low intake (OR = 1.10, 95% CI = 1.04 to 1.16), irregular nonsteroidal anti-inflammatory drugs use (OR = 1.43, 95% CI = 1.21 to 1.68) compared with regular use, and lower educational attainment (OR = 1.10, 95% CI = 1.04 to 1.16) compared with completion of at least college. The strengths of these associations were similar for late-onset CRC. As the increasing incidence of early-onset CRC was initially primarily driven by increases in rectal cancer (1), the authors also took their analysis a step further to investigate anatomic site-specific risk factors. Although low-fiber intake (per sex- and study-specific quartile) did not infer an increased risk of early-onset CRC overall, it was associated with a 24% increased risk of proximal colon cancer and a 30% increased risk of rectal cancer, but not an increased risk of distal colon cancer.

This is the largest and most comprehensive study to date attempting to elucidate the role of multiple putative risk factors for early-onset CRC. The findings lend strong support to the notion that early-onset CRC is multifactorial in nature and shares some, but not all, risk factors with later-onset CRC (9). Interestingly, mid-adulthood obesity was not associated with early-onset CRC risk, likely secondary to body mass index assessment 1-2 years prior to selection in case-control studies (8). However, a prior prospective study showed that mid-adulthood obesity, obesity at age 18 years, and weight change since age 18 years were associated with an increased risk of early-onset CRC (10). Additionally, in the current analyses, type 2 diabetes inferred a 25% nonstatistically significant elevated risk of early-onset CRC (8). Taken together, the role of obesity and metabolic dysregulation cannot be ruled out in the pathogenesis of early-onset CRC (11).

The increasing national burden of early-onset CRC has led the American Cancer Society (12) and US Preventive Services Task Force (13) to recommend initiating average-risk CRC screening at age 45 years, encompassing those at the greatest absolute risk of early-onset CRC. However, further risk-based strategies are needed to guide primary and secondary prevention. Recently, analyses from the same 3 consortia showed that individuals in the highest quartile of polygenic risk score (PRS), based on 95 genome-wide significant single nucleotide polymorphisms, had a 3.7-fold increased risk of early-onset CRC when compared with individuals in the lowest quartile (14). Earlier work from GECCO and CORECT also reported that a risk prediction model integrating family history, lifestyle factors, and PRS statistically significantly improved the accuracy of overall CRC prediction over family history alone (15). The totality of these findings demonstrates the promise of developing precision-based strategies to identify individuals at high risk for early-onset CRC and who would benefit from early prevention and detection. Although thus far the increasing incidence of early-onset CRC has been observed predominately in Whites in the United States (1), many of the putative and/or emerging risk factors disproportionally affect Blacks and Hispanics (16,17). Future research on nongenetic and genetic risk factors for early-onset CRC needs to include diverse populations to meet the demands for precision preventative strategies.

Besides screening, there is an urgent need to pool existing cohort studies/biobanks (18) that have followed participants from a young age to bolster the evidence for putative risk factors and explore predictive circulating biomarkers using prediagnostic biospecimens. Emerging risk factors (9,19) could be evaluated through real-world evidence with linkages to health records and environmental data. Novel multi-omics markers may be identified through rigorously designed studies with multiple-stage validations. Trajectories of both putative and emerging risk factors throughout the life span should be eventually incorporated to improve risk prediction based on PRS and adulthood lifestyle factors assessed at a single timepoint. Ultimately, the ways through which we elucidate the contributors to the rising incidence of early-onset CRC will pave the way to the etiologic research and prevention of other cancers that are also on the rise in the younger population. Of note, the majority of these cancers (multiple myeloma, uterine corpus, gallbladder, kidney, and pancreatic cancer) have been linked with adulthood obesity (20), and accumulating data support the role of childhood and adolescent and younger adulthood obesity in their etiopathogenesis (21–24). As such, the importance of maintaining a healthy body weight (25) and lifestyle from early childhood should not be overlooked and should be widely adopted in national and global cancer prevention strategies for immediate implementation.

## Funding

This work was supported by the National Cancer Institute at the National Institutes of Health (P30 CA091842 to GAC, R37 CA246175 to YC). EEO is supported by T32 CA009621.

## Notes

**Role of the funders:** The funders had no role in the writing of this editorial or the decision to submit it for publication.

**Disclosures:** The authors declare no conflicts of interest to disclose.

**Author contributions:** Conceptualization, EEO and YC; Writing- original draft, EEO and YC; Writing- review and editing, EEO, YC, and GAC.

## Data Availability

Not applicable.
